# Dandelion Seed Extract Affects Tumor Progression and Enhances the Sensitivity of Cisplatin in Esophageal Squamous Cell Carcinoma

**DOI:** 10.3389/fphar.2022.897465

**Published:** 2022-05-20

**Authors:** Yuxi Li, Yuying Deng, Xiuli Zhang, Han Fu, Xue Han, Wenqing Guo, Wei Zhao, Xuening Zhao, Chunxue Yu, Hui Li, Kaijian Lei, Tianxiao Wang

**Affiliations:** ^1^ School of Pharmacy, Henan University, Kaifeng, China; ^2^ Department of Botany, Liaoning Agricultural College, Yingkou, China; ^3^ School of Basic Medical Sciences, Joint National Laboratory of Antibody Drug Engineering, Henan University, Kaifeng, China

**Keywords:** dandelion seed extract, ESCC, tumor progression, cisplatin, sensitivity

## Abstract

Like dandelion, dandelion seed also have anti-inflammatory activity. Therefore, in this article, we intend to explore the anti-cancer availability of aqueous dandelion seed extract (DSE) in esophageal squamous cell carcinoma (ESCC). Firstly, the effects of DSE on cell proliferation, apoptosis, migration, invasion and angiogenesis were investigated. Then to explore the mechanism of DSE against ESCC, the levels of proliferation-associated proteins (PI3K, Akt and pAkt), apoptosis-associated proteins (survivin, Bcl-2, Bax, caspase3 and caspase9), metastasis-associated proteins (MMP2, MMP9, VEGF) and EMT progression-associated proteins (Snail, E-cadherin and Vimentin) were analyzed. Next, we further explored the effect of DSE on the sensitivity of cisplatin (DDP) in ESCC cells and investigated the effect of DSE combined with DDP on DNA damage repair-associated proteins (MSH2, MLH1 and ERCC1) and drug resistant target protein STAT3. The results indicated that DSE selectively inhibited cell growth, proliferation, migration, invasion, angiogenesis and induced cell apoptosis in ESCC cells. It was observed the decreased PI3K, Akt and pAkt proteins levels in KYSE450 and Eca109 cells administrated with DSE. The data also showed that the application of DSE decreased the level of survivin and the ratio of Bcl-2/Bax, while increased the levels of caspase3 and caspase9. We also observed that DSE significantly decreased the levels of MMP2, MMP9 and VEGF proteins and inhibited the EMT progression in KYSE450 and Eca109 cells. In addition, survivin plays a critical role in DSE against ESCC followed with the application of survivin inhibitor YM155 impairing the inhibitory abilities of DSE in ESCC cells. Meanwhile, it was observed that DSE enhances the sensitivity of DDP to human ESCC cells via promoting DNA damage and inhibiting phosphorylation of STAT3. Therefore, DSE may affect ESCC progression and enhance the sensitivity of cisplatin, and consequently become an effective anti-cancer option for human ESCC treatment.

## Introduction

Esophageal cancer is one of the commonest malignant tumors, standing fourth and sixth respectively in the mortality rate of all malignancies in China and the world. It is of concern that the incidence and mortality rate of esophageal cancer in China ranks first in the world. As the main type of esophageal cancer in China, the 5-year survival rate for Esophageal squamous cell carcinoma (ESCC) is <19% (Torre et al., 2015; Chen et al., 2016; Ghaly et al., 2016), which is due to the occurrence of metastasis in ESCC patients (Rice et al., 2017; Wang et al., 2017). So far, the mechanism of the occurrence and development of esophageal cancer is still unknown, and there is a lack of specific molecular targets and effective remedy.

Traditional Chinese medicine (TCM) plays a significant role in the adjuvant therapy of cancer. Some TCM extracts have been shown to inhibit the cells growth and metastasis of esophageal cancer (Ma et al., 2019; Shi et al., 2018; Chen et al., 2018). Dandelion (*Taraxacum* spp.) had turn into a welcome digestive remedy long before the 16th century. According to Chinese medical book, dandelion possesses the function of treating choking diaphragm, which is the most typical symptom of esophageal cancer. Previous researches have shown that dandelion exhibits anti-inflammatory and anti-cancer effects. Moreover, our previous study has shown that Dandelion root extract (DRE) significantly inhibited the growth and migration of ESCC (Duan et al., 2021). Likewise, dandelion seed has the function of antibacterial and anti-inflammatory, liver and gallbladder protection, body immunity enhancement, but its role in tumor has not been reported. Thus, to further develop the application of dandelion, we investigated the inhibitory activity of dandelion seed extract on ESCC.

The carcinogenesis of esophageal cancer is a complex process involving the accumulation and interaction of multi-factor, multi-stage and multi-gene variation. PI3K is a potential therapeutic target with the highest mutation frequency of ESCC (Wang et al., 2020; Yu et al., 2020). Survivin, Bax, Bcl-2 and caspase3/9 are apoptosis-related genes associated with esophageal cancer (Pan et al., 2015; Liu et al., 2018; Zhou et al., 2018), and MMP2/9, VEGF and EMT processes are involved in the metastasis of esophageal cancer (Li et al., 2019; Forghanifard et al., 2020; Ren et al., 2020; Tian et al., 2020). Therefore, in the present study, we detected the effects of dandelion seed extract (DSE) on the factors involving the carcinogenesis of esophageal cancer to explore the mechanism of dandelion seed extract against ESCC.

Cisplatin, as a first-line treatment for a variety of solid tumors, is used in combination with Adriamycin (ADM) and Cyclophosphamide (CTX) for esophageal squamous cell carcinoma. However, the nephrotoxicity and drug resistance of cisplatin also affect its application to some extent. Therefore, in present research, the authors investigated the sensitization of DSE to cisplatin in ESCC cells to further evaluate the function of DSE becoming an effective anti-cancer option for human ESCC treatment.

## Materials and Methods

### DSE, Positive Control Shidaopinsan (SDPS) and Cisplatin (DDP)

We purchased DSE from Huike Plant Development Co. LTD. (Shanxi, China). SDPS(Z20025080) was obtained from Conde Le Paekche pharmacy (China). We obtained DDP from Qilu Pharmaceutical Co., Ltd. (Shandong, China).

### Cell Lines

Xinxiang Medical College (Henan province, China) generously donated Human ESCC cell line KYSE450 and KYSE450 was authenticated by the Applied Biosystems in December 2019. Changzhi Medical College (Shanxi province, China)generously donated Human ESCC cell line Eca109 and Eca109 was authenticated in March 2019. Human ESCC cell lines NEC, EC9706, and TE-13 was generously donated by Xinxiang Medical College (Henan province, China). Xinxiang Medical College (Henan province, China)generously donated Normal esophageal epithelial cell line HEEPIC. The cells were maintained in Dulbecco’s modified Eagle’s medium (DMEM; Gibco; Thermo Fisher Scientific, Inc., Waltham, MA, United States) containing with 10% fetal bovine serum (FBS; Zeta Life, Inc., San Francisco, CA, United States) in a constant temperature incubator of 5% CO2 at 37°.

### Cell Viability Detection

Cell viability was detected by MTS assay. KYSE450, Eca109 and HEEPIC cells (1 × 106/ml, 100 µL/well) were seeded in 96-well plates for 24 h, then they were separately exposed to 0, 1, 2 and 4 mg/ml of DSE or SDPS for 48 h at 37°C in an atmosphere containing 5% CO2, followed by MTS addition and absorbance reading at 490 nm. Each experiment was conducted in triplicate.

### Cell Proliferation Detection

Cell proliferation was detected by 5-ethynyl-2′-deoxyuridine (EdU) assay. KYSE450 and Eca109 cells (1 × 106/ml, 100 µL/well) were plated into 96-well plates and were separately treated with 0, 1, 2 and 4 mg/ml of DSE or 4 mg/ml of SDPS at 37°C in an atmosphere containing 5% CO2 for 24 h. And then the EdU assay kit (Guangzhou Ribobio Co., Ltd., Guangzhou, China) was used to investigate cell proliferation, which was measured according to the manufacturer’s protocol.

### Cell Apoptosis Analysis

Cell apoptosis was detected by flow cytometry. Logarithmic growth cells (2 × 105 cells/well) were plated into a 6-well plate for 24 h, followed by administration of 0, 1, 2 and 4 mg/ml of DSE or 4 mg/ml of SDPS for 24 h. The cells were collected and adjusted to 5 × 105/ml by 500 µL of 1×Binding Buffer. And then added 5 µL of V-FITC and 10 µL of PI in the cell suspension, mixed gently, and stained in the dark at room temperature for 5 min. Finally, annexin V-FITC was detected by flow cytometry under 488 nm excitation and 530 nm emission, and PI was measured under 535 nm excitation and 615 nm emission.

### Wound Healing

Wound Healing was used to detect cell migration. A straight wound in cells in 35-mm dishes was scratched with a 10-μL pipette tip. After scraping 0 and 24 h, we respectively taken migration photos and compared gap closing rates.

### Transwell Assay

Cell migration and invasion was tested with transwell assay. The 24-well transwell chambers (pore size, 8 μm; Corning Incorporated, Corning, NY, United States)need to be covered with or without Matrigel matrix (BD Biosciences, CA, United States) for invasion or migration. Please refer to the literature for specific method (Duan et al., 2021).

### Tube Formation Assay

The KYSE450 and Eca109 cells were exposed to DSE for 24 h and then cultured for 24 h in serum-free medium. The culture supernatant was collected as conditioned medium. Matrigel (100 μL) was added to every well of a 24-well plate and polymerized at 37°C for 30 min. Then added HUVECs (2 × 105) in 500 μL of conditioned medium to each well and incubated in 5% CO2 for 6–8 h at 37°C. And then the cells were fixed and stained with crystal violet. We taken pictures under microscope and quantified the capillary tubes by counting number.

### Western Blot Analysis

After washed with ice-cold phosphate-buffered saline (PBS), Cells were lysed on ice using RIPA lysis buffer (Proteintech) with both protease and phosphatase inhibitors for 20 min. BCA protein assay kit (Solarbio Science & Technology Co., Ltd.) was used to determine Protein concentrations, and then Western blot analysis was carried out using a standard protocol. The primary antibodies were: PI3K rabbit polyclonal antibody (1:1,000; cat no. 20584-1-AP), Bcl-2 rabbit polyclonal antibody (1:3,000; cat no. 26593-1-AP), Caspase3 mouse monoclonal antibody (1:3,000; cat no. 66470-2-Ig), Caspase9 mouse monoclonal antibody (1:2000; cat no. 66169-1-lg), MMP2 rabbit polyclonal antibody (1:1,000; cat no. 10373-2-AP), MMP9 rabbit polyclonal antibody (1:1,000; cat no.10375-2-AP), VEGF rabbit polyclonal antibody (1:1,000; cat no. 19003-1-AP), E-cadherin rabbit polyclonal antibody (1:30,000; cat no. 20874-1-AP), Akt rabbit monoclonal antibody (1:1,000; cat no. 46853), phospho-Akt rabbit monoclonal antibody (1:1,000; cat no. 4060s), Bax rabbit monoclonal antibody (1:1,000; cat no. 5023S), Snail rabbit polyclonal antibody (1:2,000; cat no. A5243), Survivin rabbit monoclonal antibody (1:2,000; cat no. CY5070), Vimentin rabbit monoclonal antibody (1:2,000; cat no. CY5134), MLH1 rabbit polyclonal antibody (1:2,000; cat no. DF6057), MSH2 rabbit polyclonal antibody (1:2,000; cat no. DF6257), ERCC1 rabbit monoclonal antibody (1:2,000; cat no. CY6668), STAT3 mouse monoclonal antibody (1:1,000, cat. no. 9139), p-STAT3 rabbit polyclonal antibody (1:1,000, cat. no. 9131), GAPDH mouse monoclonal antibody (1:100,000; cat no. 60004-1-lg), *β*-actin mouse monoclonal antibody (1:50,000; cat no.60008-1-lg).

### Statistical Analysis

Repeated each experiment at least three times. SPSS 17.0 software (SPSS, Inc., Chicago, IL, United States) was used for data analysis. Data are expressed as the means ± standard deviation. One-way analysis of variance followed by Tukey’s Multiple Comparison Test were used to analyze differences between multiple groups. *p* < 0.05 was considered to be statistically significant.

## Results

### DSE Reduces Survival Rate and Suppresses Proliferation in Human ESCC Cells *via* Inhibiting the PI3K/Akt Pathway

**FIGURE 1:**
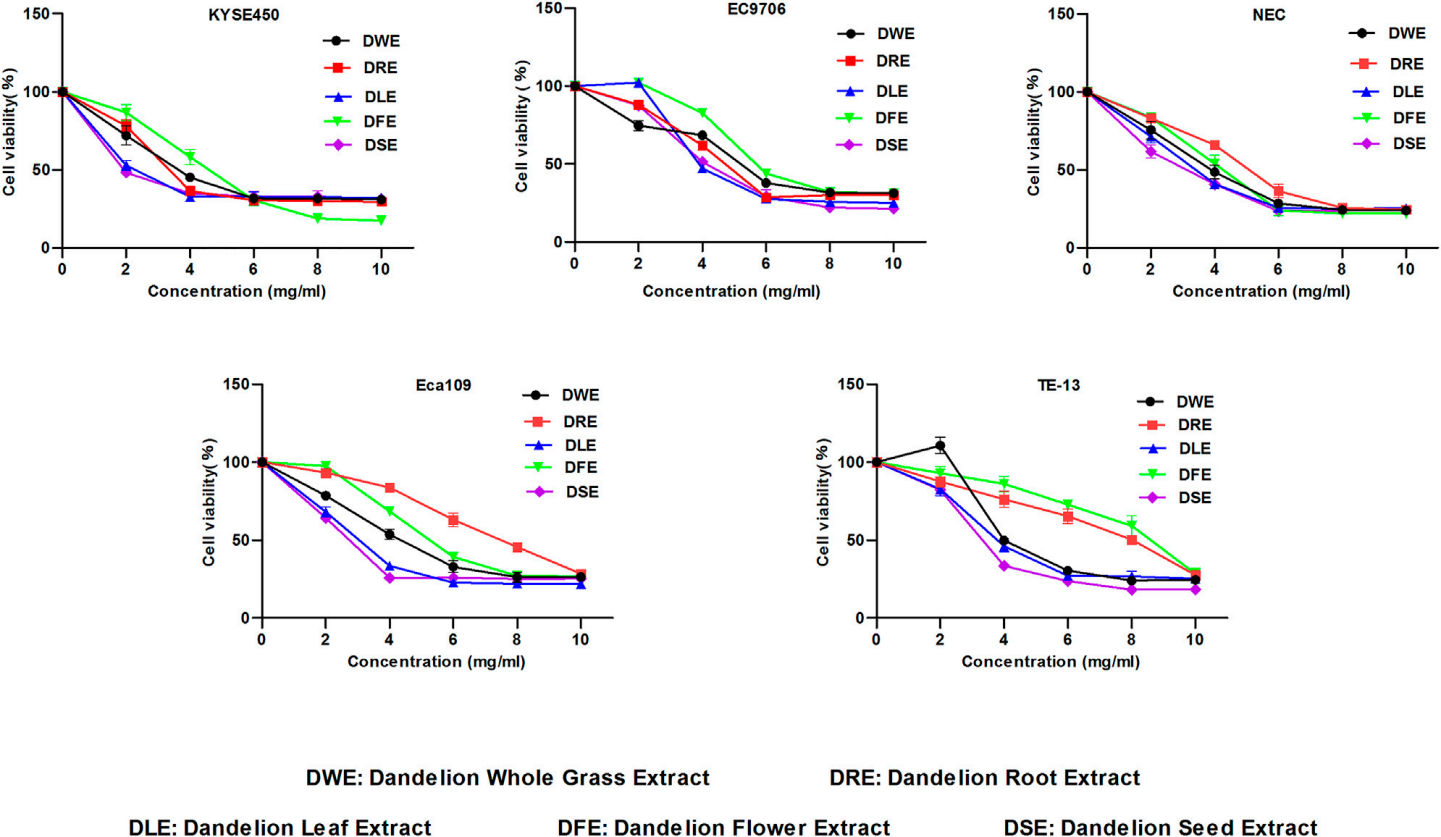
Effect of DWE, DRE, DLE, DFE and DSE on cell viability of ESCC cells. MTS assay was used to detect the cell viability of KYSE450, EC9706, NEC, Eca109 and TE-13 cells. All data are expressed as mean ± standard deviation (*n* = 3). DWE: Dandelion Whole Grass Extract; DRE: Dandelion Root Extract; DLE: Dandelion Leaf Extract; DFE: Dandelion Flower Extract; DSE:Dandelion Seed Extract.

**FIGURE 2:**
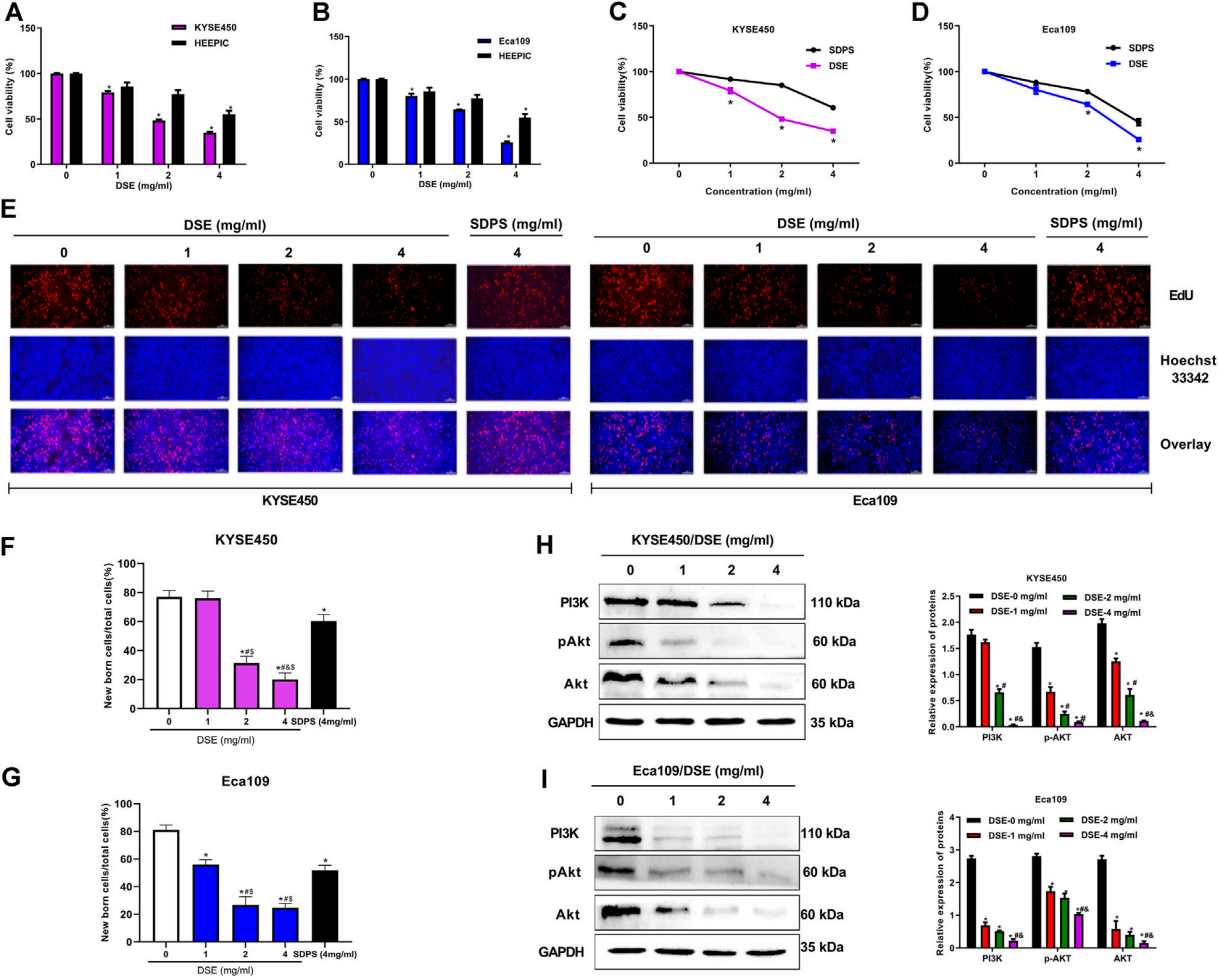
DSE suppresses cell proliferation and inhibits PI3K/Akt pathway in human ESCC cells. **(A,B)** DSE on cell viability in KYSE450, Eca109 and normal esophageal epithelial HEEPIC cells. All data are expressed as mean ± standard deviation (*n* = 3). *p < 0.05 vs. 0 mg/ml DSE group. **(C,D)** Effects of DSE and positive control SDPS on cell viability in KYSE450 and Eca109 cells. All data are expressed as mean ± standard deviation (*n* = 3). *p < 0.05 vs. SDPS group. SDPS: shidaopingsan. **(E–G)** Effects of DSE on cell proliferation in KYSE450 and Eca109 cells. Representative images were taken. Scale bars, 100 µm. All data are expressed as mean ± standard deviation (n = 3). ^*^ *p* < 0.05 vs. 0 mg/ml DSE group. ^#^ *p* < 0.05 vs. 1 mg/ml DSE group. ^&^ *p* < 0.05 vs. 2 mg/ml group. ^$^ *p* < 0.05 vs. positive control SDPS group. **(H,I)** Effects of DSE on expression of PI3K, Akt and pAkt proteins in KYSE450 and Eca109 cells. All data are expressed as mean ± standard deviation (*n* = 3). ^*^ *p* < 0.05 vs. 0 mg/ml DSE group. ^#^ *p* < 0.05 vs. 1 mg/ml DSE group. ^&^ *p* < 0.05 vs. 2 mg/ml group.

We firstly evaluated the effects of Dandelion Whole Grass Extract (DWE), Dandelion Root Extract (DRE), Dandelion Leaf Extract (DLE), Dandelion Flower Extract (DFE) and Dandelion Seed Extract (DSE) on cell viability of ESCC cells and found all extracts could inhibit the growth of ESCC to varying degrees, but DSE showed the strongest inhibitory effect (Figure 1). Therefore, we further explored the function and mechanism of DSE in ESCC in the present research. The inhibitory activity of DSE was firstly assessed in ESCC KYSE450 (IC50 = 2.223 mg/ml) and Eca109 (IC50 = 2.467 mg/ml) cells and normal esophageal epithelial HEEPIC cells. MTS results showed that DSE significantly caused a decrease in survival of ESCC in a dose-dependent manner and had lower damage to normal esophageal epithelial HEEPIC cells (Figures 2A,B). Then, we further compared the efficacy of DSE to a Chinese patent medicine shidaopinsan (SDPS) on cell survival and proliferation in KYSE450 and Eca109 cells. MTS and EdU results showed that DSE significantly inhibited the growth and proliferation of KYSE450 and Eca109 cells in a dose-dependent manner and the efficacy of DSE was significantly stronger than that of positive control SDPS (Figures 2C,G). PI3K is a potential therapeutic target with the highest mutation frequency of ESCC. PI3K/Akt pathway plays a critical role in ESCC cell proliferation (Wang et al., 2020). To investigate the mechanism of DSE against ESCC cell proliferation, we detected the effects of DSE on PI3K/Akt pathway and observed the decreased PI3K, Akt and pAkt proteins levels in KYSE450 and Eca109 cells administrated with different concentration of DSE (Figures 2H,I). These data indicate that DSE reduces survival rate and suppresses proliferation in human ESCC cells *via* inhibiting the PI3K/Akt pathway.

### DSE Induces Apoptosis of Human ESCC Cells *via* Regulating the Expression of Survivin, Bcl-2/Bax, Caspase3 and Caspase9 Proteins

**FIGURE 3:**
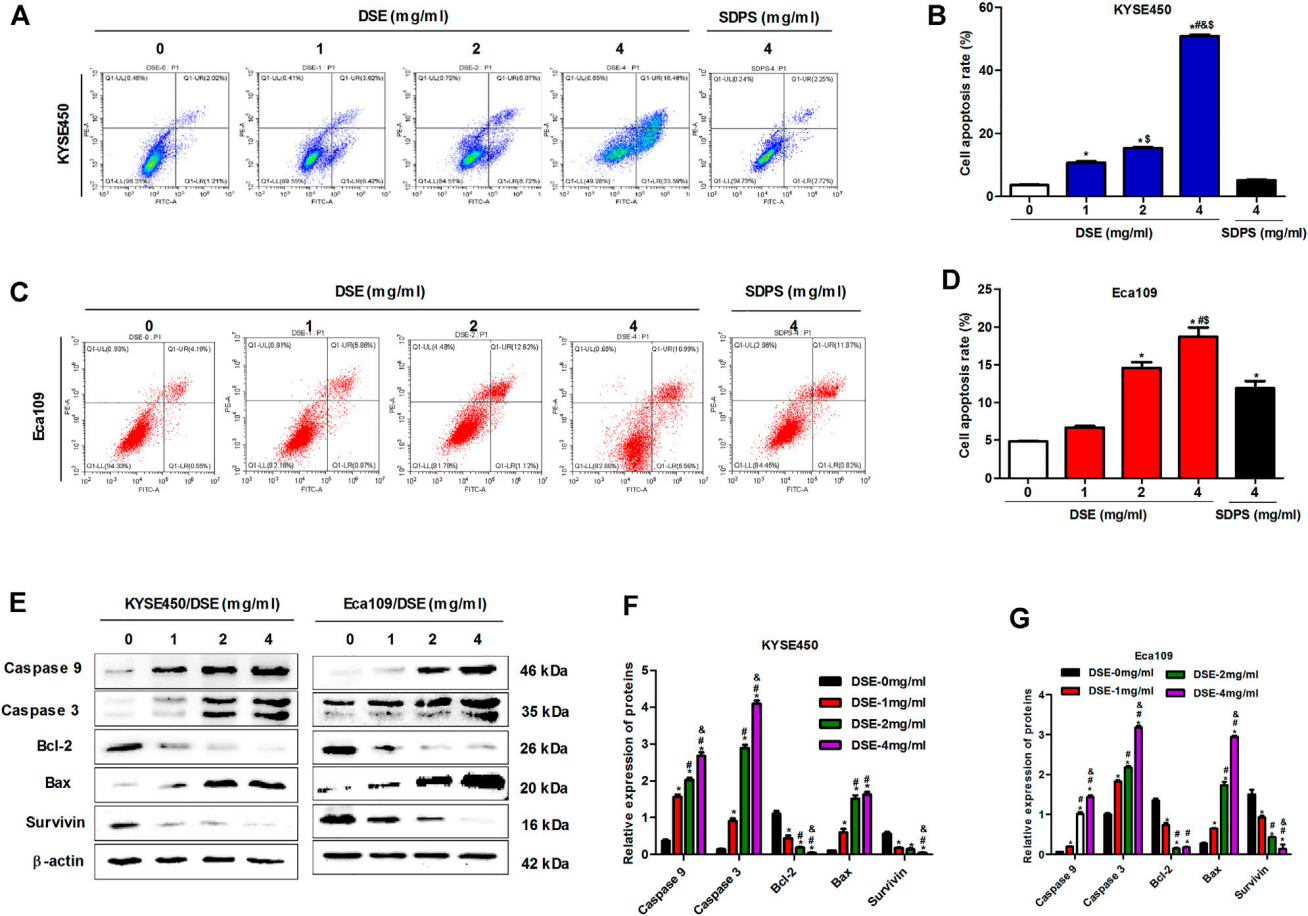
DSE induces cell apoptosis and regulates the expression of suivivin, Bcl-2, Bax, caspase3/9 proteins in human ESCC cells. **(A–D)** Effects of DSE on cell apoptosis in KYSE450 and Eca109 cells. All data are expressed as mean ± standard deviation (*n* = 3). *p < 0.05 vs. 0 mg/ml DSE group. ^#^ *p* < 0.05 vs. 1 mg/ml DSE group. ^&^ *p* < 0.05 vs. 2 mg/ml group. ^$^ *p* < 0.05 vs. positive control SDPS group. **(E–G)** Effects of DSE on the expression of suivivin, Bcl-2, Bax, caspase3 and caspase9 proteins in KYSE450 and Eca109 cells. All data are expressed as mean ± standard deviation (*n* = 3). *p < 0.05 vs. 0 mg/ml DSE group. ^#^ *p* < 0.05 vs. 1 mg/ml DSE group. ^&^ *p* < 0.05 vs. 2 mg/ml group.

Cell apoptosis inhibits oncogenesis at multiple stages. Consequently, induction of apoptosis is a critical part of tumor inhibition. Here we investigated the induction of apoptosis of ESCC by DSE and observed that DSE induced an increase in apoptosis rate of KYSE450 and Eca109 cells in a dose-dependent manner and the efficacy of DSE was significantly stronger than that of positive control SDPS (Figures 3A–D). The mechanism of DSE inducing ESCC cell apoptosis, the effects of DSE on apoptosis-related proteins were examined. The data showed that the application of DSE decreased the level of survivin and the ratio of Bcl-2/Bax, while increased the levels of caspase3 and caspase9 (Figures 3E–G), which suggested that DSE induced mitochondrial apoptosis in ESCC cells. It follows that DSE induces apoptosis of human ESCC cells and its mechanism is inducing mitochondrial apoptosis *via* regulating the expression of survivin, BCL-2/BAX, caspase3 and caspase9 proteins in ESCC cells.

### DSE Suppress Migration, Invasion and Angiogenesis of Human ESCC Cells *via* Down-Regulating MMP2, MMP9, and VEGF and Inhibiting EMT Progression

**FIGURE 4:**
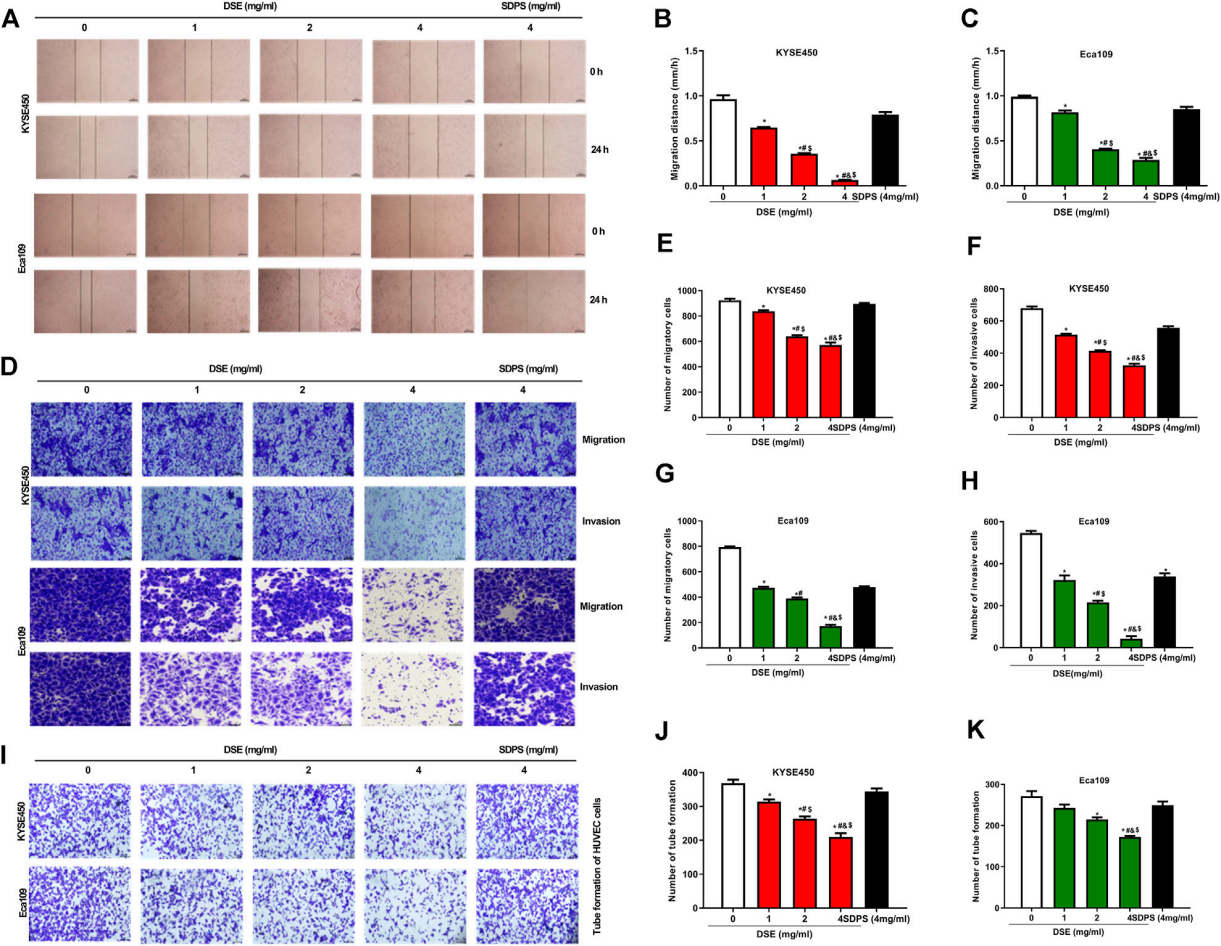
DSE inhibits migration, invasion and angiogenesis in human ESCC cells. **(A–C)** Would healing was performed to detect the effects of DSE on cell migration in KYSE450 and Eca109 cells. Representative images were taken. Scale bars, 500 µm. **(D–H)** Transwell assay was performed to detect the effects of DSE on cell migration and invasion in KYSE450 and Eca109 cells. Representative images were taken. Scale bars, 100 µm. **(I–K)** Tube formation assay was performed to detect the effects of DSE on angiogenesis induced by KYSE450 and Eca109 cells. Representative images were taken. Scale bars, 100 µm. All data are expressed as mean ± standard deviation (*n* = 3). ^*^ *p* < 0.05 vs. 0 mg/ml DSE group. ^#^ *p* < 0.05 vs. 1 mg/ml DSE group. ^&^ *p* < 0.05 vs. 2 mg/ml group. ^$^ *p* < 0.05 vs. positive control SDPS group.

To evaluate the effect of DSE on migration of ESCC cells with Wound healing assay. As shown in Figures 4A–C, DSE significantly inhibited the migration of KYSE450 and Eca109 cells in a dose-dependent manner and the efficacy of DSE was significantly stronger than that of positive control SDPS. Meanwhile, transwell assay was performed to assess the ability of ESCC cells to migrate and invade and the data showed that the number of migrating and invading cells was significantly reduced in the ESCC cells administrated with DSE (Figures 4D–H). To further investigate the angiogenesis ability of DSE in ESCC, tube formation assay was performed. We found DSE distinctly inhibited the tube formation of HUVEC cells induced by ESCC cells (Figures 4I–K). The data indicate that DSE possesses the ability to suppress migration, invasion and angiogenesis of human ESCC cells.

**FIGURE 5:**
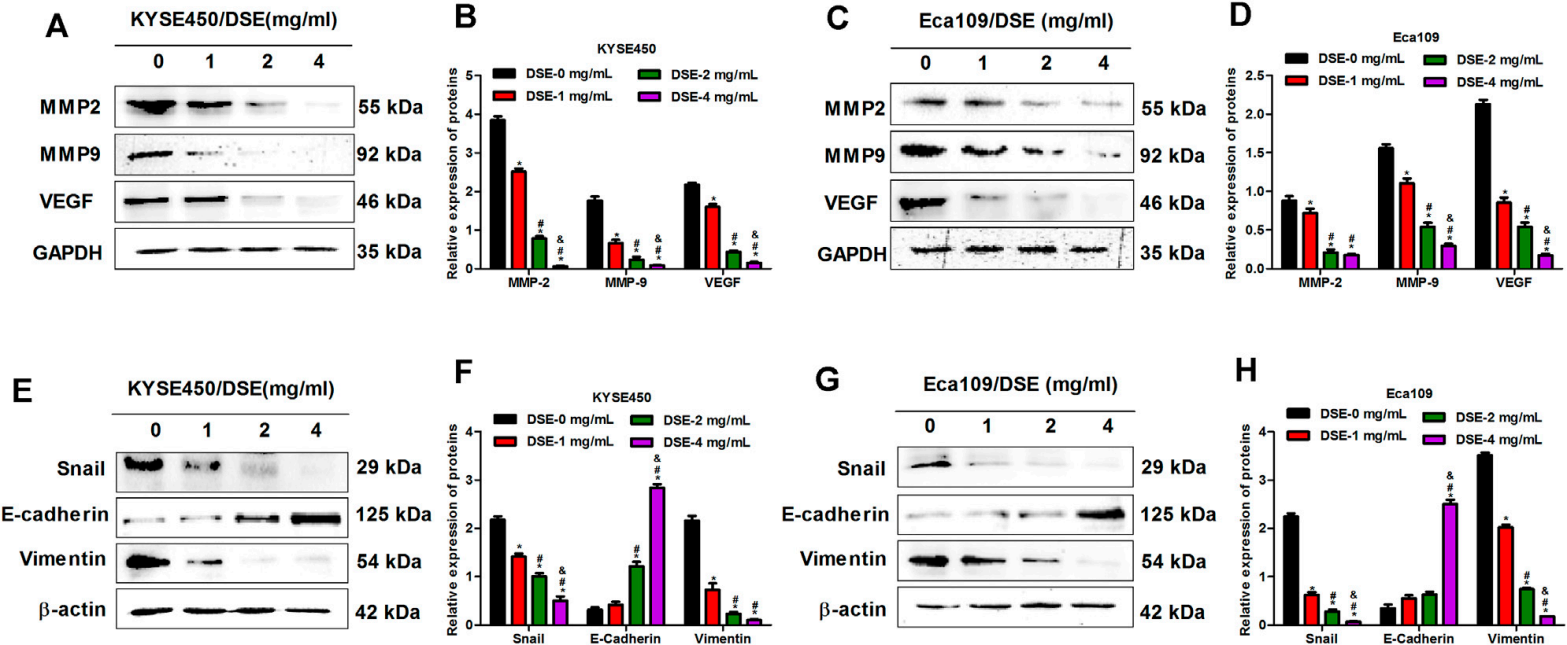
DSE down-regulates MMP2, MMP9 and VEGF levels and inhibits EMT progression in human ESCC cells. **(A–D)** Effects of DSE on the expression of MMP2, MMP9 and VEGF proteins in KYSE450 and Eca109 cells. All data are expressed as mean ± standard deviation (*n* = 3). *p < 0.05 vs. 0 mg/ml DSE group. ^#^ *p* < 0.05 vs. 1 mg/ml DSE group. ^&^ *p* < 0.05 vs. 2 mg/ml group. **(E–H)** Effects of DSE on the expression of EMT related proteins in KYSE450 and Eca109 cells. All data are expressed as mean ± standard deviation (*n* = 3). *p < 0.05 vs. 0 mg/ml DSE group. ^#^ *p* < 0.05 vs. 1 mg/ml DSE group. ^&^ *p* < 0.05 vs. 2 mg/ml group.

Matrix metalloproteinases (MMP2 and MMP9) not only release growth factors to promote the growth of tumor cells, but also promote cell shedding to facilitate the invasion and metastasis of tumor cells (Li et al., 2019; Tian et al., 2020). In addition, it can also activate VEGF to accelerate angiogenesis by initiating protein degradation of the vascular basement membrane, opening the pathway for endothelial cell metastasis to form new blood vessels (Tang et al., 2019). Therefore, we tested the effects of DSE on the expression of MMP2, MMP9 and VEGF proteins to explore the mechanism of DSE suppressing metastasis of human ESCC cells. The results indicated that DSE significantly decreased the levels of MMP2, MMP9 and VEGF proteins in KYSE450 and Eca109 cells (Figures 5A–D). Epithelial-mesenchymal transition (EMT) is an important biological process for epithelial-derived malignant tumor cells to acquire the ability to migrate and invade. EMT is characterized by up-regulation of the transcription factor Snail, decreased expression of cellular adhesion molecule E-cadherin, and cytoskeletal features dominated by Vimentin. To investigate whether DSE affects EMT process of ESCC cells, we examined the levels of Snail, E-cadherin and Vimentin proteins in KYSE450 and Eca109 cells administrated with DSE and found the treatment of DSE decreased the levels of Snail and Vimentin proteins and promoted the expression of E-cadherin protein (Figures 5E–H). The data manifest that DSE can suppress migration, invasion and angiogenesis of human ESCC cells *via* down-regulating MMP2, MMP9 and VEGF level and inhibiting EMT progression.

### Survivin Plays a Critical Role in DSE Against ESCC

**FIGURE 6:**
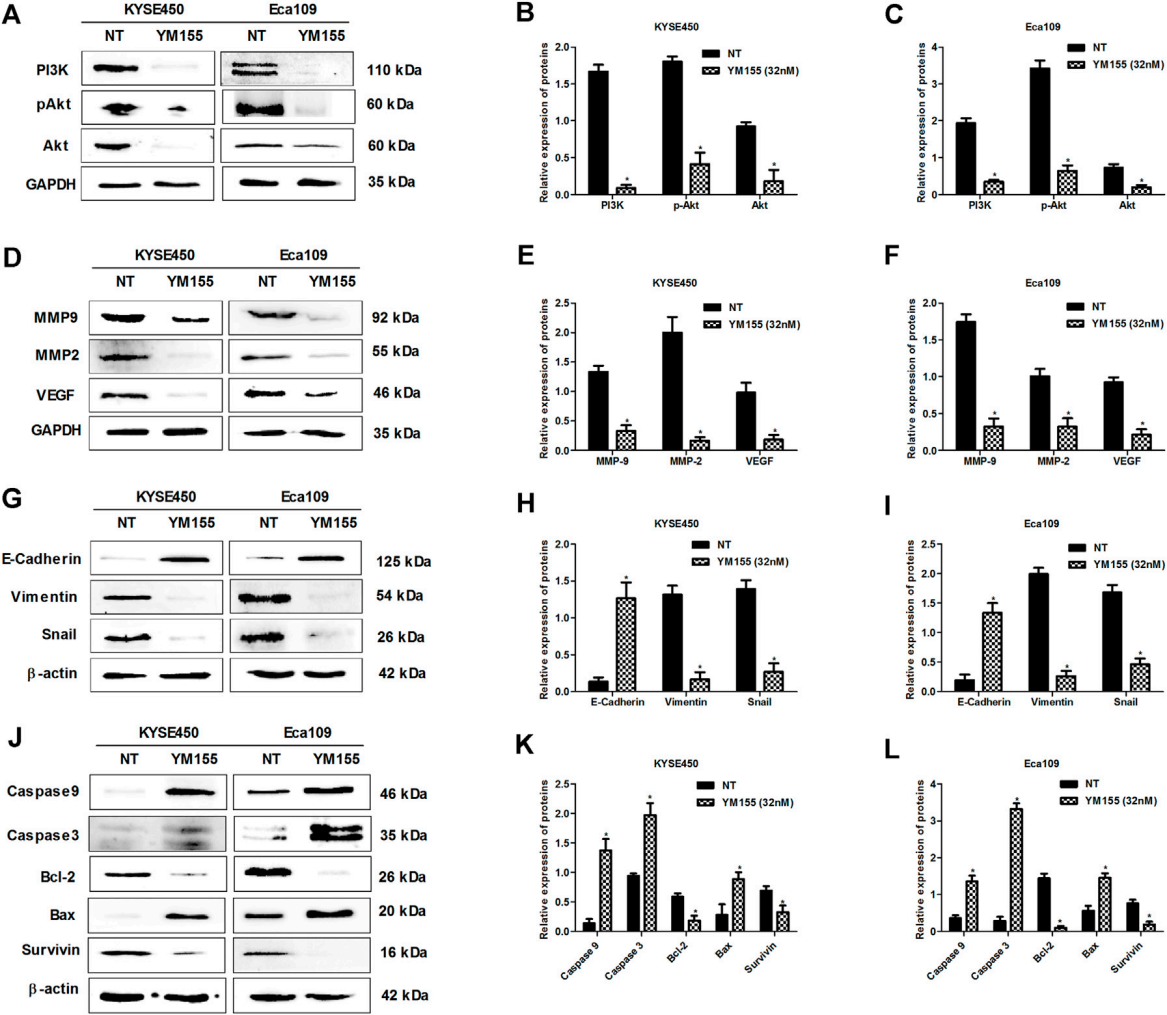
Survivin inhibitor YM155 inhibits the expression of proliferation, apoptosis and metastasis-related proteins in ESCC cells. **(A–C)** Effects of survivin inhibitor YM155 on the expression of proliferation-related proteins in KYSE450 and Eca109 cells. **(D–F)** Effects of survivin inhibitor YM155 on the expression of MMP2, MMP9 and VEGF in KYSE450 and Eca109 cells. **(G–I)** Effects of survivin inhibitor YM155 on the expression of EMT related proteins in KYSE450 and Eca109 cells. **(J–L)** Effects of survivin inhibitor YM155 on the expression of apoptosis-related proteins in KYSE450 and Eca109 cells. All data are expressed as mean ± standard deviation (*n* = 3). *p < 0.05 vs. NT group. NT: No treatment. YM155 concentration: 32 nM.

**FIGURE 7:**
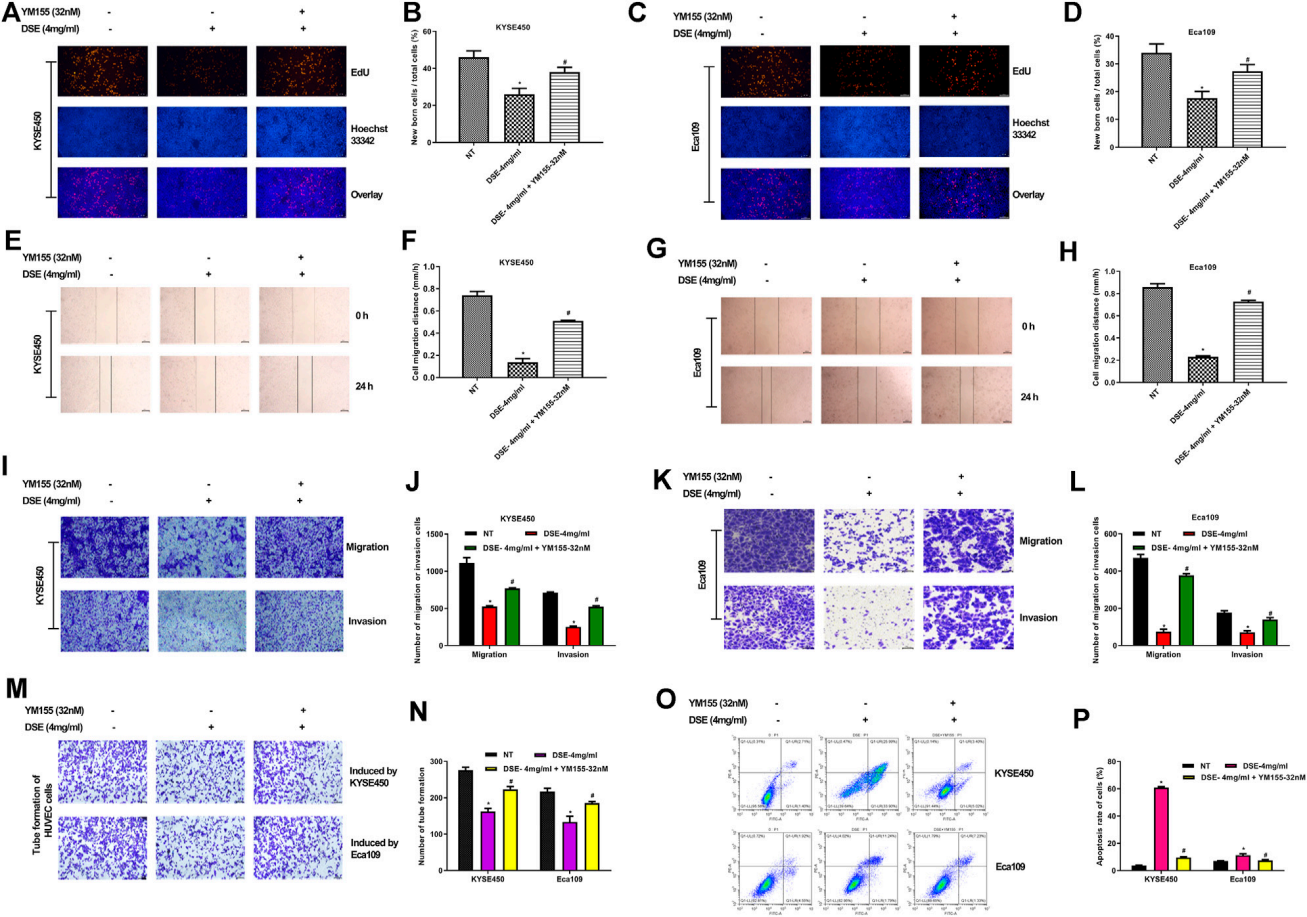
Survivin inhibitor YM155 impairs the abilities of DSE in ESCC cells. **(A–D)** Effects of survivin inhibitor YM155 on proliferation inhibition induced by DSE in KYSE450 and Eca109 cells. Representative images were taken. Scale bars, 100 µm. **(E–H)** Effects of survivin inhibitor YM155 on migration inhibition induced by DSE in KYSE450 and Eca109 cells. Representative images were taken. Scale bars, 500 µm. **(I–L)** Effects of survivin inhibitor YM155 on migration and invasion inhibition induced by DSE in KYSE450 and Eca109 cells. Representative images were taken. Scale bars, 100 µm. **(M,N)** Effects of survivin inhibitor YM155 on angiogenesis inhibition induced by DSE in KYSE450 and Eca109 cells. Representative images were taken. Scale bars, 100 µm. **(O,P)** Effects of survivin inhibitor YM155 on apoptosis induced by DSE in KYSE450 and Eca109 cells. All data are expressed as mean ± standard deviation (*n* = 3). *p < 0.05 vs. NT group. ^#^ *p* < 0.05 vs. DSE group. NT: No treatment.

Previous researches have indicated that survivin is overexpressed in esophageal cancer cells and the expression of survivin sustains growth and confers resistance to chemotherapy in esophageal cancer cells (Hui et al., 2012; Zhou et al., 2018; Yan et al., 2020), which suggests survivin plays a significant role in esophageal cancer. In present research, we also found that survivin inhibition affected the expression of above-mentioned proteins related to proliferation (PI3K, Akt, pAkt), apoptosis (Bcl-2, Bax, Caspase3/9) and metastasis (MMP2, MMP9, VEGF, Snail, E-cadherin, Vimentin) in ESCC (Figure 6), which further demonstrated that survivin plays a significant role in ESCC. To further investigate the effect of survivin on DSE against esophageal squamous cell carcinoma, survivin inhibitor YM155 was firstly used to treat the ESCC cells and then the effects of DSE on proliferation, apoptosis, migration, invasion and tube formation ability were detected. The data showed that the application of survivin inhibitor YM155 impaired the abilities of DSE in proliferation inhibition (Figures 7A–D), migration or invasion inhibition (Figures 7E–L), angiogenesis inhibition (Figures 7M,N) and apoptosis induction (Figures 7O,P)in ESCC cells, which indicated that survivin plays a critical role in DSE against ESCC.

### DSE Enhances the Sensitivity of DDP to Human ESCC Cells

**FIGURE 8:**
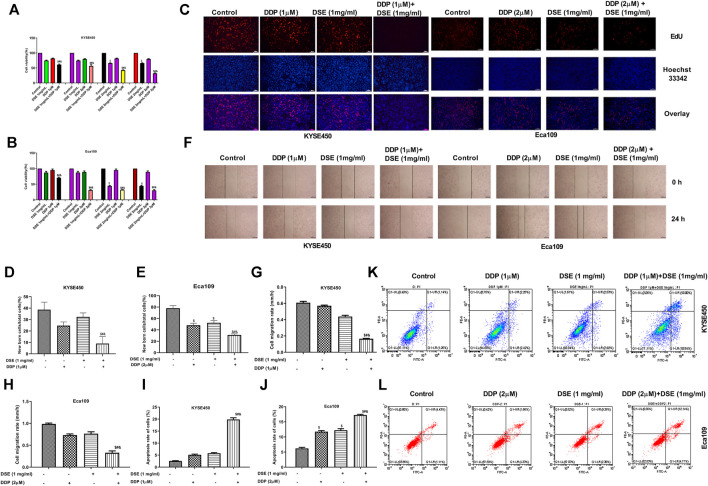
DSE enhances the sensitivity of DDP to human ESCC cells. **(A,B)** Effects of combination of DSE and DDP on the survival rate of KYSE450 and Eca109 cells. **(C–E)** Effects of combination of DSE and DDP on the proliferation of KYSE450 and Eca109 cells. Representative images were taken. Scale bars, 100 µm. **(F–H)** Effects of combination of DSE and DDP on the migration of KYSE450 and Eca109 cells. Representative images were taken. Scale bars, 500 µm. **(I–L)** Effects of combination of DSE and DDP on the apoptosis of KYSE450 and Eca109 cells. All data are expressed as mean ± standard deviation (*n* = 3). ^$^ *p* < 0.05 vs. control group. ^#^ *p* < 0.05 vs. DSE group. ^&^ *p* < 0.05 vs. DDP group.

**FIGURE 9:**
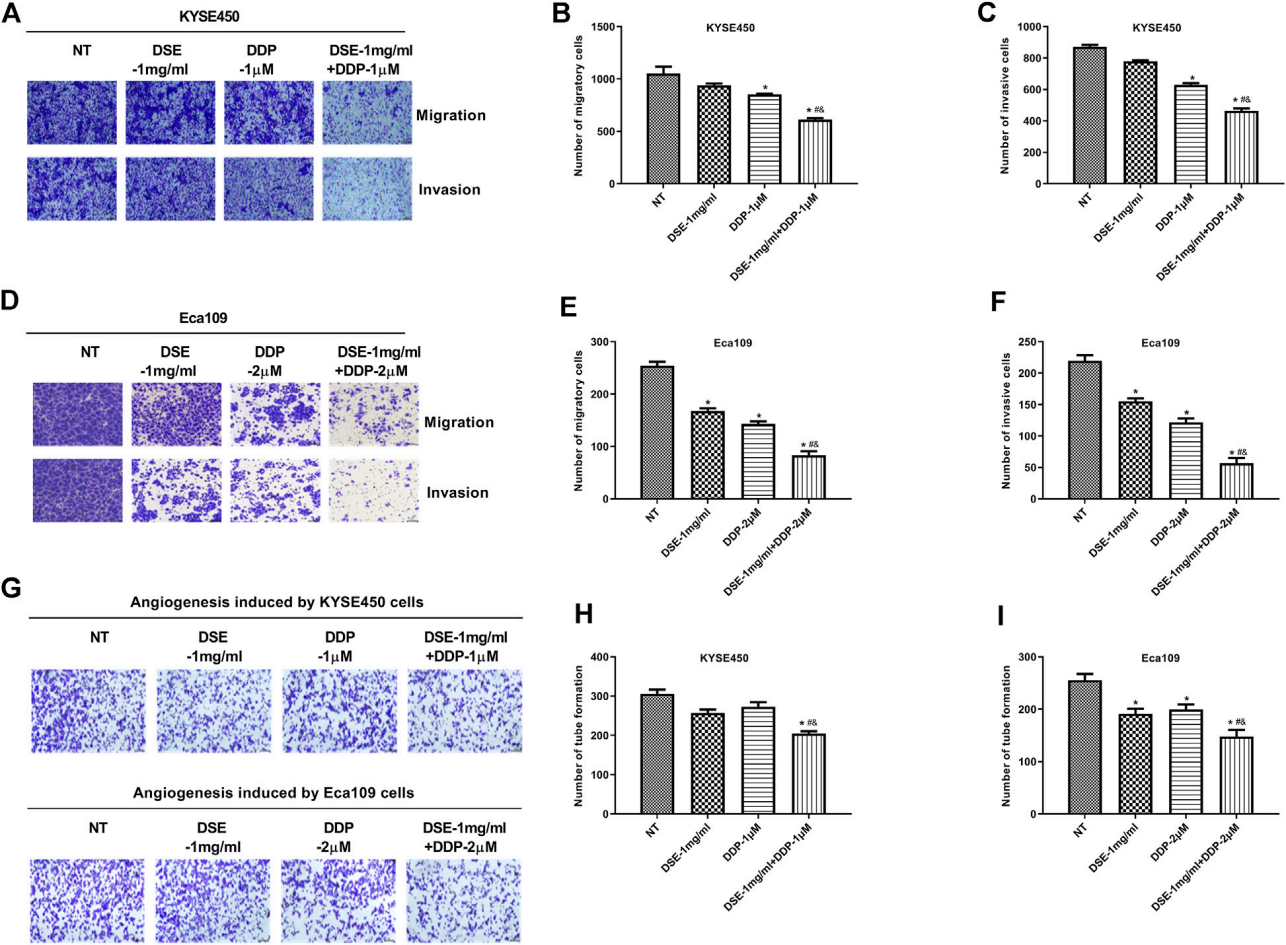
DSE combined with DDP inhibits migration, invasion and angiogenesis of human ESCC cells. **(A–C)** Effects of the combination of DSE and DDP on migration and invasion of KYSE450. **(D–F)** Effects of the combination of DSE and DDP on migration and invasion of Eca109 cells. **(G–I)** Effects of the combination of DSE and DDP on angiogenesis of KYSE450 and Eca109 cells. Representative images were taken. Scale bars, 100 µm. All data are expressed as mean ± standard deviation (*n* = 3). *p < 0.05 vs. NT group. ^#^ *p* < 0.05 vs. DSE group. ^&^ *p* < 0.05 vs. DDP group. NT: No treatment.

The above results indicate that DSE may be a potential therapeutic agent for ESCC. Next, we further explored whether DSE can enhance the effect of cisplatin (DDP) in ESCC cells. The effects of DSE combined with DDP on the proliferation, apoptosis, migration, invasion and tube formation ability of ESCC were detected. The data showed that the combination of DSE and DDP enhanced the sensitivity of DDP, which showed decreased cell survival rate (Figures 8A,B) and proliferation ability (Figures 8C–E) as well as migration ability (Figures 8F–H) and increased apoptosis rate (Figures 8I–L), compared with DDP or DSE alone. Meanwhile, transwell assay and tube formation assay were also performed to further evaluate DSE combined with DDP in esophageal squamous carcinoma metastasis inhibition. The results indicated that the combination of DSE and DDP decreased the number of migration and invasion cells (Figures 9A–F) and inhibited angiogenesis induced by esophageal squamous cancer cells (Figures 9G–I), compared with DDP or DSE alone. All data suggest that DSE enhances the sensitivity of DDP to human ESCC cells.

### DSE Promotes DNA Damage and Inhibits Phosphorylation of STAT3

**FIGURE 10:**
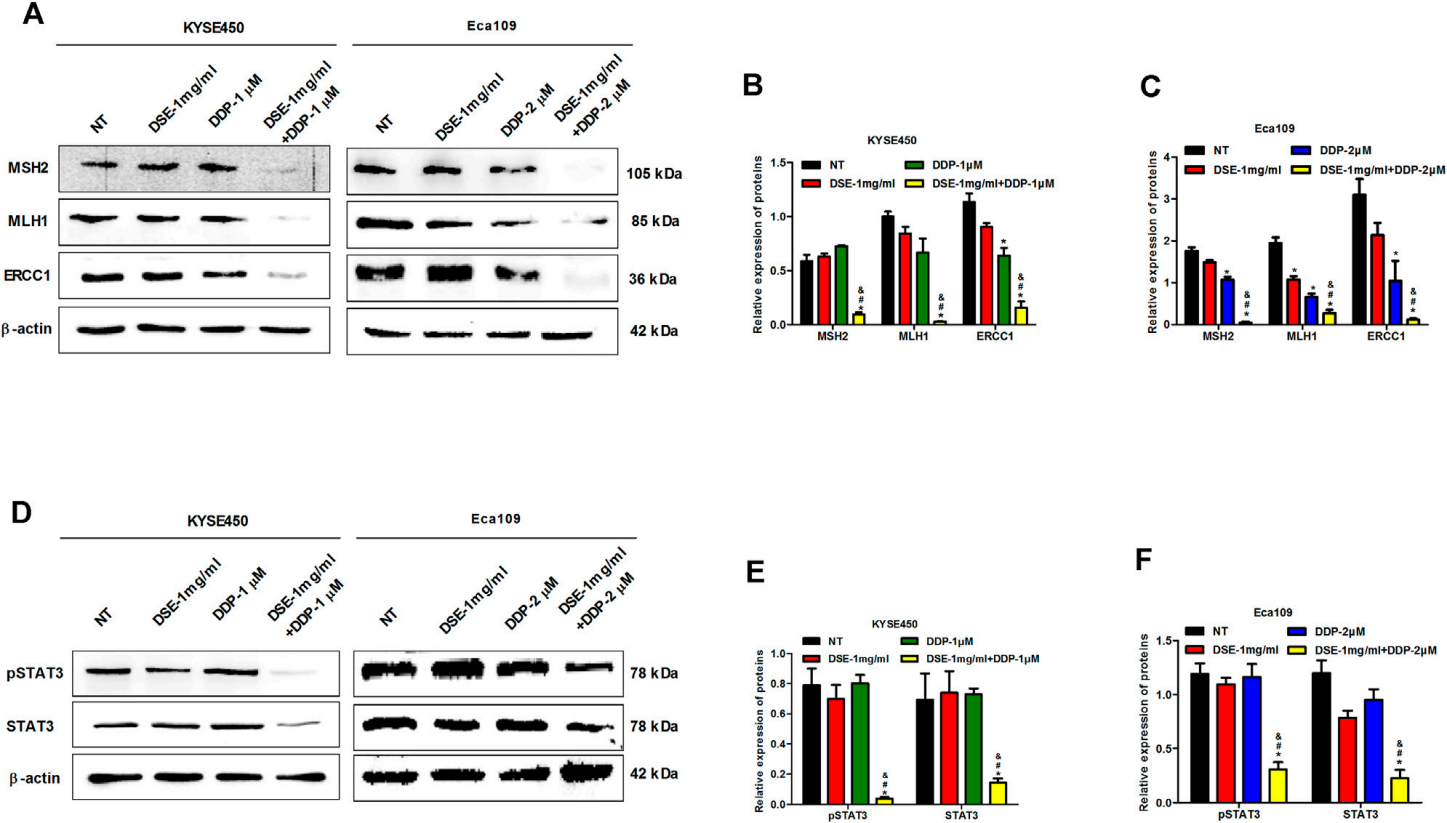
DSE promotes DNA damage and inhibits phosphorylation of STAT3. **(A–C)** Effects of the combination of DSE and DDP on the expression of DNA repair-related proteins MSH2, MLH1 and ERCC1 in KYSE450 and Eca109 cells. **(D–F)** Effects of the combination of DSE and DDP on the expression of STAT3 and pSTAT3 in KYSE450 and Eca109 cells. All data are expressed as mean ± standard deviation (*n* = 3). *p < 0.05 vs. NT group. #*p* < 0.05 vs. DSE group. ^&^ *p* < 0.05 vs. DDP group. NT: No treatment.

Mismatch repair proteins-MSH2 and MLH1 and excision repair cross complement (ERCC) are involved in DNA damage repair and consequently related to the sensitivity of cisplatin in cancer. Therefore to explore the mechanism of DSE to enhance the sensitivity of DDP in ESCC, we investigated the effects of DSE combined with DDP as well as DDP or DSE alone on MSH2, MLH1 and ERCC1 expression. The authors found that the combination of DSE and DDP significantly reduced the level of MSH2, MLH1 and ERCC1 proteins, compared with DDP or DSE alone (Figures 10A–C), which indicated that DSE promoted DNA damage and consequently enhanced the sensitivity of DDP in ESCC. In addition, transcription factor STAT3, as the target protein drug resistance, is also associated with the sensitivity of DDP in cancer. Here, we observed that the combination of DSE and DDP distinctly inhibited the phosphorylation of STAT3 in KYSE450 and Eca109 cells, compared with DDP or DSE alone (Figures 10D–F), which suggested that DSE could inhibit the activation of STAT3 to enhance the sensitivity of DDP.

## Discussion

Like dandelion, dandelion seeds also have anti-inflammatory activity. Previous studies have shown that dandelion exhibits anti-cancer activities. However, whether dandelion seeds have anti-cancer activity is unknown. The present study explores the anti-cancer potential of dandelion seeds extract (DSE) in esophageal squamous cell carcinoma (ESCC). Our data showed that DSE significantly inhibited the growth, proliferation, migration, invasion and angiogenesis and induced the apoptosis in ESCC cells. The results indicate that DSE possesses the ability to resist ESCC.

PI3K is a potential therapeutic target with the highest mutation frequency of ESCC and plays a vital role in ESCC cell proliferation (Wang et al., 2020). Matrix metalloproteinases facilitate the invasion and metastasis of tumor cells and can activate VEGF to accelerate angiogenesis (Li et al., 2019; Tian et al., 2020). Meanwhile, The important biological process for epithelial-derived malignant tumor cells to acquire the ability to migrate and invade is EMT (Forghanifard et al., 2020; Ren et al., 2020). Therefore, to explore the possible mechanisms of DSE against proliferation and metastasis of ESCC cells, we tested the effects of DSE on PI3K/Akt pathway, the expression of MMP2, MMP9 and VEGF proteins and EMT progression. We found that DSE reduced survival rate and suppressed proliferation *via* inhibiting the PI3K/Akt pathway in human ESCC cells. The data also demonstrated that the down-regulation of MMP2, MMP9 and VEGF proteins and inhibition of EMT progression were involved in DSE-mediated metastasis in ESCC cells. In addition, the data also demonstrated that DSE induced apoptosis of human ESCC cells *via* regulating the expression of survivin, the ratio of Bcl-2 and Bax, and the levels of caspase3 and caspase9 proteins.

Previous studies showed that the expression of survivin sustains growth and confers resistance to chemotherapy in esophageal cancer cells (Hui et al., 2012; Zhou et al., 2018; Yan et al., 2020). Therefore, survivin plays a significant role in esophageal cancer. In present study, we also further demonstrated that survivin plays a significant role in ESCC because survivin inhibition affected the expression of proteins related to proliferation, apoptosis and metastasisin ESCC. Moreover, we found that the application of survivin inhibitor YM155 impaired the inhibitory abilities of DSE in ESCC cells, which suggested that survivin played a critical role in DSE against ESCC.

Cisplatin is a first-line treatment for a variety of solid tumors and is used in combination with ADM and CTX for esophageal squamous cell carcinoma. However, the nephrotoxicity and drug resistance of cisplatin also affect its application to some extent. Therefore, the development of synergistic and attenuating agents is very vital, and the adjuvant therapy of Traditional Chinese Medicine may play an important role. In present study, we explored that the effects of DSE combined with DDP on the proliferation, apoptosis, migration, invasion and tube formation ability of ESCC cells and found that DSE enhanced the sensitivity of DDP to human ESCC cells.

Next, to further explore the mechanism of DSE to enhance the sensitivity of DDP in ESCC, we investigated the effects of DSE combined with DDP on DNA repair related proteins MSH2, MLH1 and ERCC1 expression. The authors found that the combination of DSE and DDP significantly reduced the levels of MSH2, MLH1 and ERCC1 proteins, and consequently promoted DNA damage and enhanced the sensitivity of DDP in ESCC. In addition, the combination of DSE and DDP distinctly inhibited the phosphorylation of STAT3 in ESCC cells, which was associated with the sensitivity of DDP in cancer.

In conclusion, the present study indicated that aqueous DSE effectually inhibits cell growth in ESCC and confirmed that survivin is responsible for DSE anti-cancer activity. At the same time, DSE can enhance the sensitivity of DDP to human ESCC cells *via* promoting DNA damage and inhibiting phosphorylation of STAT3. So, we can conclude that DSE, as a complex mixture, might provide a complementary option to currently available chemotherapies and serve as a potential effective anti-cancer alternative for human ESCC therapies. Thus, further assessment of the efficacy of DSE would be performed *in vivo* (animals) in the future work.

## Data Availability

The original contributions presented in the study are included in the article/Supplementary Material, further inquiries can be directed to the corresponding authors.
